# Generation and Characterization of an IgG4 Monomeric Fc Platform

**DOI:** 10.1371/journal.pone.0160345

**Published:** 2016-08-01

**Authors:** Lu Shan, Magali Colazet, Kim L. Rosenthal, Xiang-Qing Yu, Jared S. Bee, Andrew Ferguson, Melissa M. Damschroder, Herren Wu, William F. Dall’Acqua, Ping Tsui, Vaheh Oganesyan

**Affiliations:** 1 Antibody Discovery and Protein Engineering, MedImmune, Gaithersburg, Maryland, United States of America; 2 Clinical Pharmacology and Drug Metabolism and Pharmacokinetics, MedImmune, Gaithersburg, Maryland, United States of America; 3 Analytical Biotechnology, MedImmune, Gaithersburg, Maryland, United States of America; 4 Discovery Sciences, Structure and Biophysics, AstraZeneca Pharmaceuticals, Waltham, Massachusetts, United States of America; Saint Louis University, UNITED STATES

## Abstract

The immunoglobulin Fc region is a homodimer consisted of two sets of CH2 and CH3 domains and has been exploited to generate two-arm protein fusions with high expression yields, simplified purification processes and extended serum half-life. However, attempts to generate one-arm fusion proteins with monomeric Fc, with one set of CH2 and CH3 domains, are often plagued with challenges such as weakened binding to FcRn or partial monomer formation. Here, we demonstrate the generation of a stable IgG4 Fc monomer with a unique combination of mutations at the CH3-CH3 interface using rational design combined with *in vitro* evolution methodologies. In addition to size-exclusion chromatography and analytical ultracentrifugation, we used multi-angle light scattering (MALS) to show that the engineered Fc monomer exhibits excellent monodispersity. Furthermore, crystal structure analysis (PDB ID: 5HVW) reveals monomeric properties supported by disrupted interactions at the CH3-CH3 interface. Monomeric Fc fusions with Fab or scFv achieved FcRn binding and serum half-life comparable to wildtype IgG. These results demonstrate that this monomeric IgG4 Fc is a promising therapeutic platform to extend the serum half-life of proteins in a monovalent format.

## Introduction

**Fig 1:**
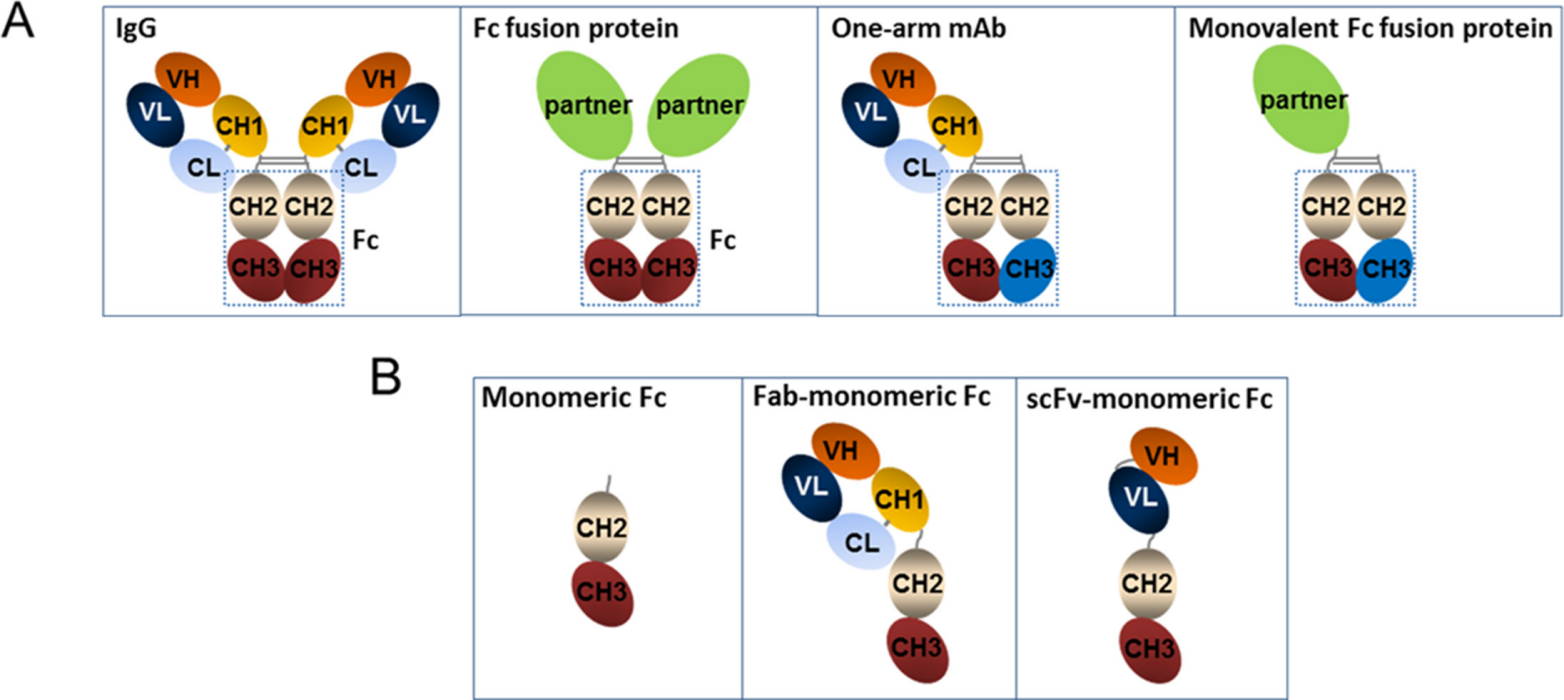
Cartoon representations of wildtype IgG Fc, monomeric Fc and fusion proteins. (**A**) Cartoons of Fc homodimer in IgG and in a bivalent Fc fusion protein, as well as a one-arm mAb and a monovalent Fc fusion, supported by heterodimeric Fc (as shown) or tethered Fc. (**B**) Cartoons of a monomeric Fc, along with Fab- and scFv- fusion proteins with a monomeric Fc.

The homodimeric immunoglobulin fragment crystallizable, Fc, has been widely utilized to form fusion proteins with enzymes, growth factors, immune modulators, and target-binding moieties such as scFv [1–4] (**Fig 1A**). Both as research tools and as therapeutic agents, Fc-fusion proteins are able to harness FcRn-mediated serum half-life extension provided by the Fc domain. In recent years, there have been several examples of proteins fused on one arm of the Fc, e.g., erythropoietin, coagulation factor IX, and interferon, that exhibited similar or improved stability and biological activities compared to conventional Fc fusions [5–8]. In addition, there are particular signaling pathways, such as receptor tyrosine kinases, which require monovalent targeting to avoid receptor agonism caused by receptor dimerization from bivalent antibodies or Fc fusions [6].

Monovalent versions of Fc fusion proteins (Alprolix—coagulation factor IX fusion, Eloctate—factor VIII fusion) or monovalent antibodies (Onartuzumab—anti-cMet one-arm mAb) that have advanced to the clinic utilize an Fc domain that is engineered to form a heterodimer, either with tethering or “knobs-into-holes” technology [7, 9]. These, along with other heterodimeric Fc technologies, rely on robust purification processes to remove undesired chain pairing and achieve a homogeneous fusion protein [10] (**Fig 1A**). To search for an alternative approach aimed at simplifying product development, there has been extensive effort in engineering fusion protein platforms with a monomeric Fc modality consisted of only one set of CH2 and CH3 domains (**Fig 1B**), either through weakening the interactions or by generating steric hindrances with the addition of glycans at the CH3-CH3 dimer interface in the Fc [11–13]. So far these approaches have encountered challenges in several aspects, including solubility and stability, loss of FcRn binding, or lack of homogeneity. Additionally, many of the previously engineered monomeric Fc molecules were observed by dynamic light scattering to have a tendency for aggregation, highlighting the challenge of stabilizing the monomeric conformation after weakening the homodimer interface [12, 14]. To date, the only available crystal structure of monomeric Fc has been the glycoengineered Fc monomer, where an additional glycan at the dimer interface resulted in a stable monomer [11]. There has also been some evidence that avidity of the bivalent Fc has a large contribution to FcRn binding [11]. This suggests that monomeric Fc, without additional half-life extension technology, would result in dramatic loss of binding to FcRn [11, 12]. To compensate for the lower FcRn binding affinity, linking monomeric Fc in tandem format has been utilized [11], which further complicates the biophysical characteristics of the final fusion molecules.

We report here the development of a therapeutic platform for the expression of a monomeric Fc fusion protein that exhibits FcRn binding affinity comparable to the wildtype Fc. We devised a comprehensive protein engineering approach that involved using a unique IgG4 phage library design and thermal stability and folding selections, in addition to a pH-dependent FcRn binding selection, to identify a monomeric Fc with excellent monodispersity. Our results show that a library selection strategy combining thermal selection and rational template designs can lead to monomeric Fc fusion proteins which have the desired biophysical, structural and pharmacokinetics (PK) properties.

## Materials and Methods

### Ethics statement

Protocol (MI-13-0012) requiring the use of animals in these studies was reviewed and approved by MedImmune’s Institutional Animal Care and Use Committee and complies with the animal welfare standards of the USDA, Guide for the Care and Use of Laboratory Animals, and AAALAC international.

### Reagents

Restriction enzymes and polymerases were purchased from New England Biolabs (Ipswich, MA) and Clontech (Mountain View, CA). Oligonucleotides were purchased from Integrated DNA Technologies (Coralville, Iowa). Recombinant human and murine FcRn were expressed and purified as described previously [15]. Antibody positions are listed according to the Kabat numbering convention [16].

### Phage library design, selection and screening

An IgG4 Fc template containing M252Y/S254T/T256E (YTE) and F405R was constructed in a phage vector [15]. A phage library was built with random mutations at five additional CH3-CH3 interface residue positions, L351, S354, T366, P395 and Y407 [13, 17, 18]. Based on previous work showing Fc had favored monomer formation when mutated to R, Q or E from F at position 405, we fixed these three mutations in the library build [12, 13, 19]. Five primers were designed to cover the positions, L351/S354, T366, P395, Y407/F405RQ, Y407/F405E, respectively, to yield 3 overlapping PCR fragments, which were then annealed together. The library insert was ligated into the phage vector. The library diversity was achieved at 1×10^9^ covering all possible mutations.

Human FcRn was expressed in human embryonic kidney cell line HEK293F(Life Technologies, Grand Island, NY) and purified as a soluble protein [15]. Library selection by biopanning against FcRn generally followed previously described procedures [15], with some modifications. Library phages were first passed through a Protein G column (Roche) to eliminate phages displaying misfolded Fc mutants which had lost Protein G binding [13]. For each round of the pH-dependent FcRn binding selection, amplified libraries of 10^12^ phages were biopanned against biotinylated FcRn. The libraries and streptavidin-coated magnetic DynaBeads (Thermo Fisher Scientific, Waltham, MA) were first blocked with 20% bovine serum albumin (BSA) at pH 6.0 for 1 hour. A deselection step was then carried out by mixing blocked beads and the libraries to remove non-specific bead-binding phages. Then the deselected libraries were incubated with 75 nM of biotinylated FcRn and the blocked beads at pH 6.0 for 2 hours. Extensive washes were carried out on KingFisher instrument (Thermo Fisher). The phages were eluted with phosphate buffered saline (PBS, 5 mM sodium phosphate and 155mM NaCl), 0.01% Tween-20, at pH 7.4. The eluted phages were grown and amplified using *Escherichia coli* TG1 cells (Lucigen, Middleton, MI) and prepared for the next round of selection. Starting at round 3 of library selection, library selection steps were performed with and without thermal stress. Thermal stress was carried out by elevating the phage incubation temperatures from 37 to 50°C in rounds 3 and 4, and further increased to 55°C in round 5 [20]. Forty eight clones were picked from rounds 4 and 5 of the library selection output and were expressed by inducing with 1 mM IPTG (isopropyl-1-thio-β-D-galactopyranoside, Sigma-Aldrich, St. Louis, MO) for 3 hours at 37°C. The bacterial lysates were analyzed by Western blotting under non-reducing conditions. Phages from each clone were prepared by infecting TG1 cells with helper phage MK13KO7 (Life Technologies) to be screened by FcRn binding ELISA at pH 6.0 and pH 7.4. Neutravidin at 5 μg/mL in PBS (pH 6.0) was coated on a Nunc Maxisorp Plate wells (Thermo Fisher) overnight at 4°C. After three washes, biotinylated FcRn was added at 2 μg/mL, and the wells were blocked with 4% BSA in PBS buffer with 0.1% Tween 20. Phages diluted in 1% BSA in the same buffer were added and incubated for 1 hour. The plates were then washed three times and anti-M13-HRP antibody (GE Healthcare, Pittsburgh, PA) incubated for 1 hour. Binding was detected with the addition of SureBlue 3,3′,5,5′-tetramethylbenzidine (TMB) substrate (KPL, Gaithersburg, MD), and the reaction was stopped by adding the TMB Stop Solution (KPL). Absorbance signals were read at 450 nm. Phages showing positive binding to FcRn in ELISA were sequenced and subsequently cloned into a proprietary mammalian vector for expression and purification.

### Cloning, expression and purification of anti-cMet monomeric Fc fusion proteins

Four FcRn binding clones identified from the library selection were used for mammalian expression. They were purified using Protein A column and further characterized. After confirming its monomeric state, clone 4 (C4) was used to generate monomeric Fc fusion proteins with the Fab (Onart-Fab-C4) and the scFv (Onart-scFv-C4) of the antibody Onartuzumab [6].

### Size-exclusion chromatography with light scattering and analytical ultracentrifugation

Purified Fc clones and fusion proteins at 1 mg/mL or higher were analyzed by size exclusion chromatography using a TSK-GEL G2000SWXL column with a 14 mL bed volume (Tosoh Biosciences, Tokyo, Japan) on an Agilent 1100 HPLC (Agilent, Santa Clara, CA) at room temperature. The samples were eluted isocratically in PBS at a flow rate of 1 mL/min for 20 min. Eluted proteins were detected using UV absorbance at a wavelength of 280 nm. Data analysis was done using the Agilent software ChemStation (version A.02.10). Column calibration was performed with a set of molecular weight standards ranging from 10 to 500 kDa (Bio-Rad, Hercules, CA). In-line multi-angle light scattering (SEC-MALS) was performed. Sample measurements were done on a DAWN HELEOS II MALS with an Optilab Rex refractometer (Wyatt Technologies, Santa Barbara, CA). Molecular mass of each protein within a defined chromatographic peak was calculated using ASTRA, version 6.1 (Wyatt Technologies). For analytical ultracentrifugation analysis, samples and reference buffer were loaded into 12 mm double-sector cells with Epon centerpieces then placed in an An-50 rotor for ultracentrifugation at 42,000 rpm using a Beckman Optima XL-I centrifuge set to 20°C (Beckman-Coulter, Indianapolis, IN). The sedimentation data collected at 280 nm for scans 2 to 140 were analyzed using the Sedfit software program (version 14.6e) to generate c(s) distributions [21, 22]. The partial molar volume was set to a value of 0.73 g/mL. Solution density and viscosity values for PBS were set to 1.00523 g/mL and 1.019 mPa·s, respectively, using the calculated value from the Sednterp program (version 20130813) [23]. Based on the Svedberg equation a monomeric Fc with molecular mass of 28 kDa is expected to have a sedimentation coefficient of between 1.8 to 2.6 S (Svedbergs) assuming a frictional ratio of 1.3 to 1.8 (globular to extended shape).

### Crystallization, data collection and structure determination

Prior to crystallization, Protein A-purified C4n (clone C4 with **n**o YTE) was further purified by ion exchange chromatography on a Q HP 5 mL prepacked column (GE) equilibrated with 25 mM HEPES buffer at pH 7.5, followed by size exclusion chromatography using a Sepharose 75 column (GE) pre-equilibrated with 25 mM HEPES buffer with 100 mM NaCl at pH 7.5. Isocratically eluted protein was concentrated to 4 mg/mL and subjected to crystallization using commercial crystallization screens in a sitting drop format with Phoenix (ARI, Mountain View, CA). Diffraction quality crystals were grown in the crystallization optimization step in a hanging drop format from a solution containing 9 mM ZnCl_2_, 90 mM HEPES, 18% PEG 6000 (w/v) at pH 7.0. Cryoprotection was achieved by transferring crystals through several solutions of increasing glycerol concentrations, up to 20% v/v, added to the crystal growth solution. Crystals were flash cooled by dipping in liquid nitrogen. Complete diffraction data to 1.95 Å were collected on LS-CAT beamline 21-ID at Argonne National Laboratory (Lamont, IL) using x-ray wavelength 1.00Å and oscillation range of 0.5°. Seven hundred twenty images were collected using Mar300 CCD detector and processed using HKL2000 [24]. All crystallographic calculations were carried out using the CCP4 software suite (version 6.5) [25]. The molecular replacement procedure was carried out using Molrep [26]. Structure refinement was performed using Refmac5 and model adjustments were carried out using the “O” program [27, 28]. Images for the structure figures were generated using PyMOL (Schrödinger, LLC).

### Octet binding analysis

Binding measurements of the monomeric Fc and its fusion proteins to in-house purified recombinant human and murine FcRn, FcγRI, FcγRIIIa and an inactive form of IgG-degrading enzyme of *S*. *pyogenes* (IdeS, C94S) [29] were carried out by Bio-layer Interferometry on an Octet384 instrument (ForteBio, Menlo Park, CA). Biotinylated FcRn at 1 μg/mL in PBS buffer (pH 7.4) or 100 mM MES buffer (pH 6.0), with 3 mg/ml BSA, 0.05% (v/v) Tween 20 (1× Kinetics Buffer, ForteBio) were captured on streptavidin (SA) biosensors (ForteBio). The loaded biosensors were washed with assay buffer to remove any unbound protein, followed by association and dissociation measurements with serial dilutions of the different Fc variants or Fc-fusion constructs. Kinetic parameters (k_on_ and k_off_) and apparent affinities (K_D_) were calculated from a non-linear fit based on the 1:1 binding kinetic model $\left(A+B\underset{k_{off}}{\overset{k_{on}}{↔}}\;AB\right)$ of the data using the Octet384 software (v.7.2). Kinetic measurements of anti-cMet fusion proteins to recombinant human cMet protein were also performed. Fc-fusion proteins were captured on anti-human Fc (AHC) biosensors (ForteBio) in PBS buffer, pH 7.2, with 1× kinetics buffer. Binding steps were done with serial dilutions of cMet.

### cMet activity assays

Cell viability was measured by the CellTiter-Glo® Luminescent Cell Viability Assay (Promega, Madison, WI) kit. Lovo cells were trypsinized, washed and seeded at 100 μl in 96-well plates at a density of 1 × 10^4^ cells/well in serum-free cell media with 0.1% BSA. After 2-hour incubation at 37°C in 5% CO_2_ atmosphere, 50 μl of antibodies at various concentrations in triplicates were added to the cells and incubated for another hour. Then 50 μl of human growth factor (HGF) was added to the ligand-containing wells at the final concentration of 20 ng/mL. The plates were incubated for 72 h at 37°C. After ligand treatment, the cells were exposed to the CellTiter-Glo® reagent for 20 min and the luminescence was measured using an EnVision plate reader (PerkinElmer, Waltham, MA). The IC_50_ values were defined as the antibody concentrations inhibiting cell growth by 50%.

### *In vivo* PK studies in hFcRn transgenic mice

hFcRn transgenic mice used in this study are the F1 cross of murine FcRn-deficient B6.129X1-*Fcgrt*^*tm1Dcr*^/DcrJ and hFcRn cDNA transgenic line B6.Cg-*Fcgrt*^*tm1Dcr*^ Tg (CAG-FCGRT) 276 Dcr/DcrJ [30, 31]. Sex-matched (6–16-week-old) mice were given a bolus intravenous dose of 2.5 mg/kg monomeric Fc fusion proteins on day 0. Eight mice were used per protein, with two groups of mice (A group or B group) bled at alternate time points. Blood samples were obtained from the retro-orbital plexus using capillary pipettes at different time points throughout the 2–3-week long study. All animals remained healthy throughout the study. A quantitative ELISA was used to monitor the serum concentrations of the tested antibodies. Briefly, 96-well plates were coated with 2 μg/ml cMet-ECD (extracellular domain). Plates were blocked with 3% BSA in PBS at pH 7.2 for 1 hour and then incubated with appropriately diluted serum samples. Goat anti-human Fc-specific HRP-conjugated antibody (dilution 1:5000; Jackson ImmunoResearch Laboratories, West Grove, PA) was used for detection. Absorbance at 450 nm was measured after development with 3,3′,5,5′-tetramethylbenzidine substrate (KPL) according to the manufacturer's directions. Standard curves were generated for each antibody variant diluted into 1:100 prebleed mouse sera taken at day −3. The linear portions of standard curves generated in Prism (Version 6, GraphPad Software, La Jolla, CA) were then used to quantify human anti-cMet fusion proteins in the serum samples.

### PK data analysis

Non-compartmental PK data analysis was performed using Phoenix 64 WinNonlin 6.3 (Pharsight, Mountain View, CA). The maximum observed peak plasma concentration (C_max_) was determined by inspection of the observed data using WinNonlin. The terminal elimination half-life (t_½_) was determined using the equation ln(2)/λz, where λz is the slope of the terminal portion of the natural-log concentration-time curve, determined by linear regression of at least the last 3 time points. The systemic exposure was determined by calculating the area under the curve for the plasma concentration versus time graph (AUC_last_) from the start of dosing to the time of last measurable concentration using the linear/log trapezoidal rule. Area under the curve for the plasma-concentration vs. time graph from time 0 to infinity (AUC_∞_) was calculated as: AUC_last_ + C_last_/λz, where C_last_ is the last quantifiable concentration. Clearance (CL) was calculated by dose/AUC_∞_, and steady state volume of distribution (V_ss_) was calculated as: (AUMC_∞_×CL)/AUC_∞_, where AUMC_∞_ is the area under the curve from the first moment extrapolated to infinity. PK parameters were summarized statistically and presented as mean and one standard deviation.

## Results

### Design and screening for monomeric IgG4 Fc

**Fig 2:**
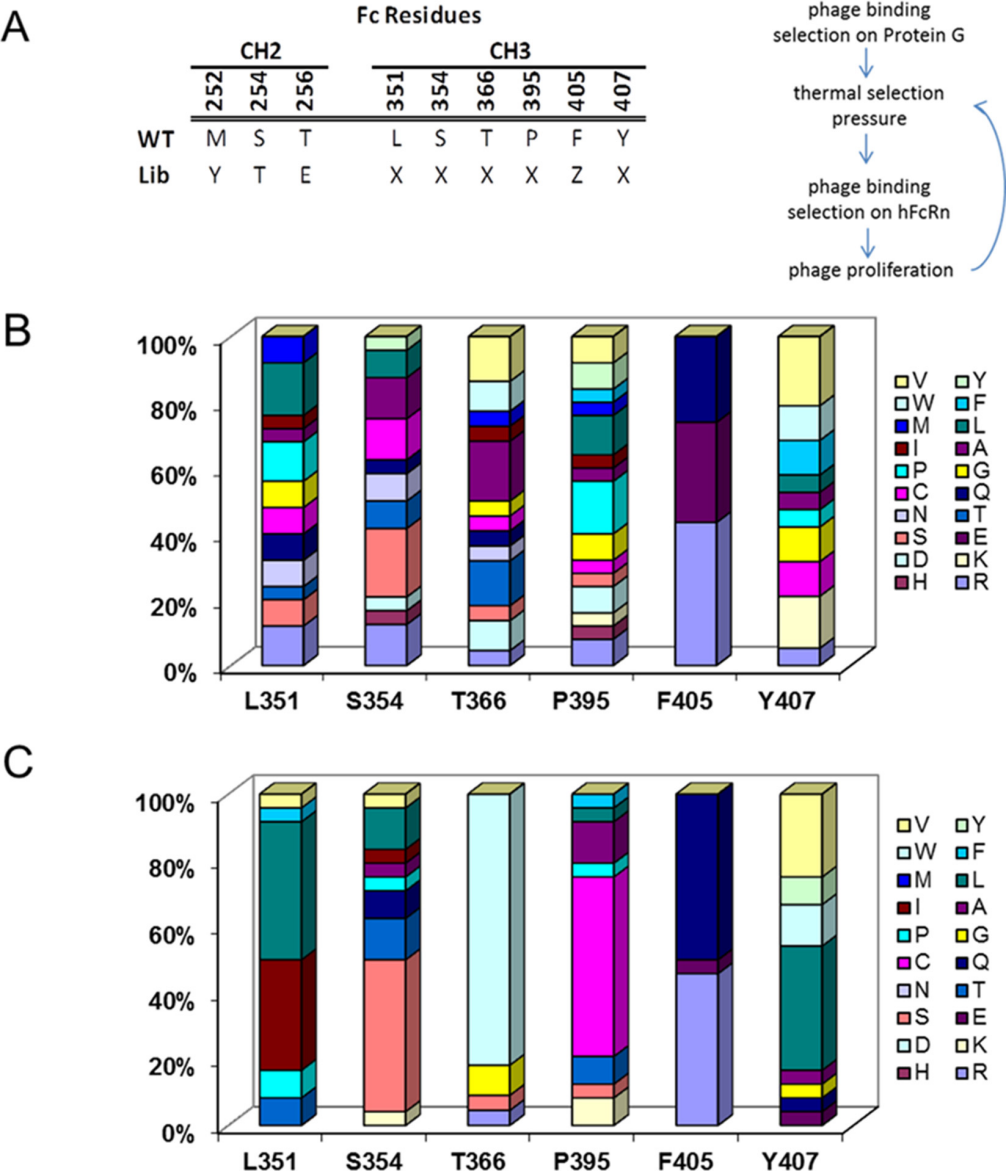
Monomeric Fc library design, selection workflow and output analysis. (**A**) Phage display library with mutations in CH2 and CH3 was built as indicated in the sequence alignment. X represents any amino acid and Z represents R, E or Q. The library selection was carried out first with Protein G binding, then several iterative rounds of thermal stress and FcRn binding. At the end of the library selection, randomly picked clones were sequenced and the frequency of amino acids at each position, in single letter codes, was plotted in the bar graphs. Parallel biopanning rounds with no thermal stress (**B**) and with thermal stress (**C**) showed the improved enrichment under thermal selection pressure.

The Fc homodimer interface is a well packed region between two CH3 domains mediated by over 16 residues in all of the IgG subclasses [13, 17, 18]. In particular, IgG4 is unique in its ability to form monomer-dimer equilibrium, partially due to the contribution of the K409R point mutation compared to IgG1-3 [32, 33]. Previous work with scanning mutagenesis gave valuable insight for key interface residues in IgG4 that contributed to the homodimer formation [12]. Based on these findings, we designed a phage library to maximize the chance to identify an IgG4 monomeric Fc with improved FcRn binding affinity, homogeneity and stability (**Fig 1B**), by fully exploring the sequence combinations at the CH3-CH3 interface positions, L351, S354, T366, P395 and Y407, with random mutagenesis. We fixed point mutations F405R/E/Q in the library build, which had been shown previously to be favored in a monomeric state [12, 13, 19]. In addition, YTE mutations (M252Y/S254T/T256E), known for IgG half-life extension via tighter FcRn binding, were placed in the library template, so that any selected final sequence will be compatible with these residues (**Fig 2A**).

To increase the selection pressure on more stable monomeric clones, we incorporated a protein G binding selection and a thermal challenge to the library phages, in addition to the pH-dependent FcRn binding selection (**Fig 2A**) [20]. The phages were incubated at 50°C during rounds 3 and 4 of biopanning, and at 55°C in round 5. Whereas very little amino acid enrichment was seen in the biopanning rounds against FcRn binding without thermal selection, the biopanning done with thermal selection was able to achieve notable enrichment (**Fig 2B and 2C**). For position 351, the mutations narrowed down to residues L, I, F, T and V. Position 354 was enriched to residues S, T and L. Position 366 has the most enrichment to W, followed by G, R and S. Position 395 is preferentially selected to more polar residues (C, E and K). The three F405 mutations to Q, E and R in the library template were seen to have narrowed down R and Q. Tyrosine residue at position 407 is changed to V and L, and to a smaller extent, polar residues (E and H).

### Characterization of library-derived clones

**Fig 3:**
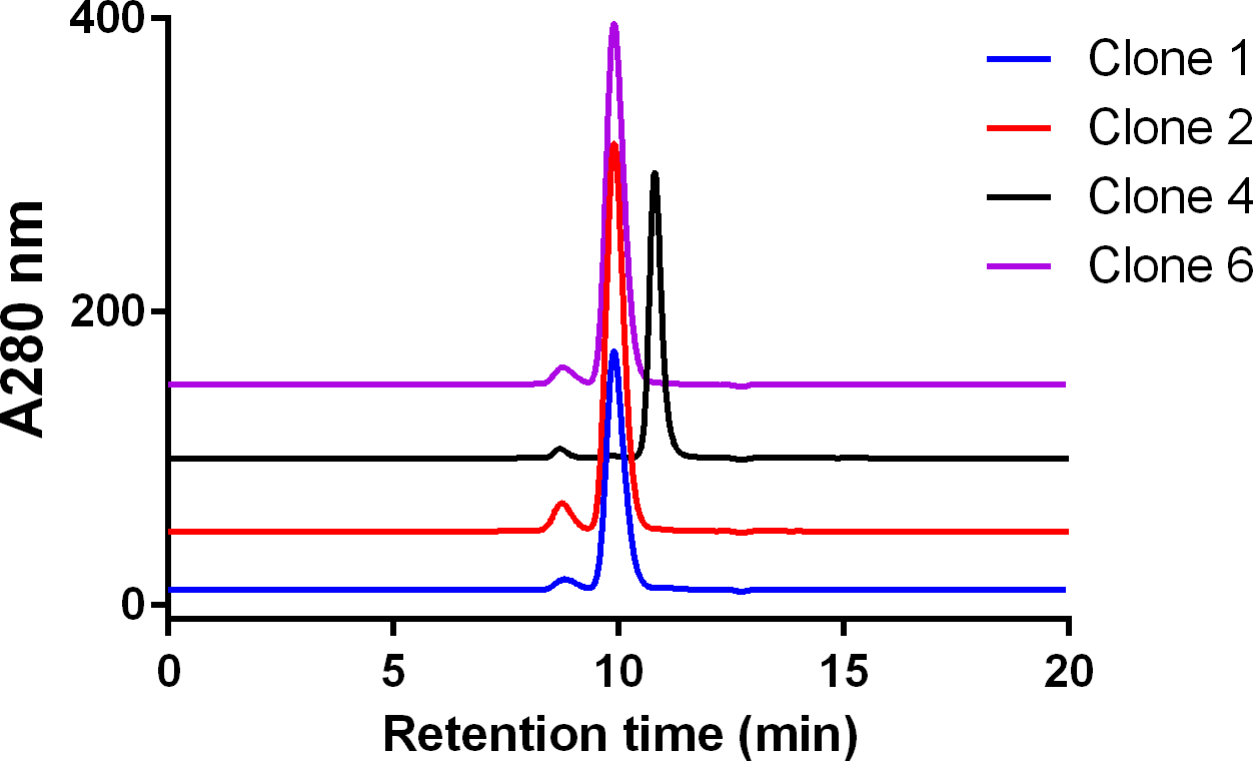
Analytical size-exclusion chromatograms of purified lead Fc clones 1, 2, 4 and 6. The retention times of clones 1, 2 and 6 appear shorter than that of clone 4, suggesting a difference in their molecular masses.

Clones derived from the thermal selection were screened for FcRn binding using phage ELISA. Four FcRn binding clones 1, 2, 4 and 6 were selected to express corresponding proteins using mammalian cells. Purified proteins were analyzed by SEC-MALS. All the mutants showed good homogeneity with clones 1, 2 and 6 showing slightly shorter retention times in the SEC profiles (**Fig 3**). The MALS data revealed significantly different molecular weights for these clones (**Table 1**). Clone 4 (C4) showed the expected molecular mass of ~27 kDa, confirming its monomeric state, which was further confirmed by analytical ultracentrifugation, with a measured sedimentation coefficient of 2.2 S (expected monomer sedimentation coefficient is between 1.8 and 2.6 S). Analysis of C4 and C4n (C4 without YTE) at high concentrations (>10 mg/mL) by SEC found that the proteins remained monomeric (data not shown). Aside from the YTE mutations, the mutations at the CH3-CH3 dimer interface in C4 were L351F, T366R, P395K, F405R, and Y407E. Interestingly, clones 1, 2 and 6, which did not show monomeric characteristics in SEC and MALS, had common mutations L351I, T366W and Y407L, possibly resulting in increased hydrophobicity at the interface. Only C4 was carried forward as a monomeric Fc to generate fusion proteins to achieve better FcRn binding characteristics with the YTE mutations, while C4n was used to determine the crystal structure to present the monomeric Fc in a more general way (without YTE).

**Table 1:** Molecular weights of Fc fragments and fusion proteins determined by MALS.

Construct	Mn (g/mol)	Mp (g/mol)	Mw (g/mol)	Mw/Mn
Clone 4	2.7×10^4^ (±0.8%)	2.7×10^4^ (±0.8%)	2.7×10^4^ (±0.8%)	1.001 (±1.2%)
Clone 1	5.7×10^4^ (±0.3%)	5.7×10^4^ (±0.2%)	5.7×10^4^ (±0.3%)	1.000 (±0.4%)
Clone 2	5.3×10^4^ (±0.3%)	5.3×10^4^ (±0.1%)	5.3×10^4^ (±0.3%)	1.001 (±0.4%)
Clone 6	5.3×10^4^ (±0.2%)	5.4×10^4^ (±0.1%)	5.3×10^4^ (±0.2%)	1.000 (±0.3%)
Onart-Fab-C4	7.7×10^4^ (±0.9%)	7.6×10^4^ (±0.9%)	7.7×10^4^ (±0.9%)	1.000 (±1.2%)
Onart-scFv-C4	5.9×10^4^ (±0.9%)	5.8×10^4^ (±0.9%)	5.9×10^4^ (±0.9%)	1.001 (±1.3%)

Mn represents number-average molar mass. Mp represents molar mass at concentration peak. Mw represents weight-average molar mass. Mw/Mn represents polydispersity.

### Structure determination and refinement

The monomeric Fc mutant C4n formed sizeable rhombohedral shape crystals that diffracted to a resolution of 1.95 Å. The morphology of crystals corresponded to their symmetry R32 (SG #155). In a hexagonal setting (H32) the cell parameters are a = b = 121.4 Å, c = 99.7 Å. The asymmetric part of the unit cell contained one polypeptide with Matthew’s parameter of 2.7 Da/Å^3^ with a solvent content of 55%. The structure was solved by molecular replacement. As a template for molecular replacement, a 1.9 Å resolution IgG4 Fc model from PDB ID 4C54 was used [34]. For most of the molecule, the initial electron density maps were of excellent quality, allowing for the placement of correct amino acids at the mutation sites. The X-ray data and refinement statistics are shown in **Table 2**.

**Table 2:** X-ray data and model refinement statistics.

**Data statistics**	
Wavelength (Å)	1.0000
Resolution (Å)	46.50–1.95 (2.02–1.95)*
Space group	H32
Unit-cell parameters (Å)	a = 121.4, b = 121.4, c = 99.7
Total reflections	229813 (20424)
Unique reflections	19638 (2038)
Completeness (%)	99.9 (100.0)
R_merge_	0.064 (0.718)
Mean *I*/*σ* (*I*)	33.1 (2.2)
Multiplicity	11.1 (11.3)
**Refinement statistics**	
Resolution (Å)	24.00–1.95
R_work_/R_free_/R_work+free_	0.199/0.259/0.202
RMSD bonds (Å)	0.018
RMSD angles (°)	2.041
Ramachandran outliers (%)	0.0
Number of protein atoms	1679
Number of non-protein atoms	199
Mean B factor (Model/Wilson) (Å^2^)	47.5/50.0

*Values in parentheses correspond to the highest-resolution shell

**Fig 4:**
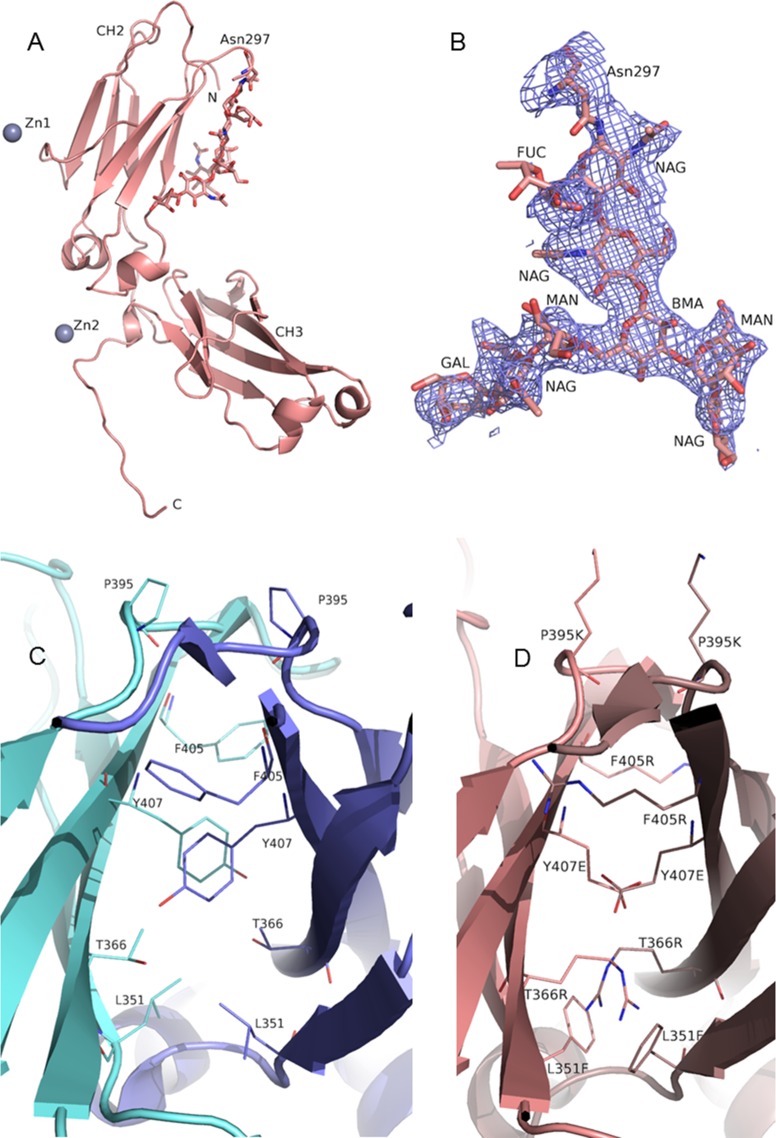
Cartoon representation of the X-ray crystal structure of monomeric Fc C4n. (**A**) The protein chain and carbohydrates attached to N297 are shown in light salmon color. Light blue spheres indicate Zn atoms. (**B**) The model of the carbohydrate moiety attached to N297 superimposed onto the electron density map. Both of the possible terminal sialic acids and one of the galactose residues have no corresponding electron density map. The fucose attached to the core GlcNAc showed only partial electron density. (**C**) CH3-CH3 interface of wildtype IgG4 Fc showing side chains of the amino acids targeted in the mutagenesis library. Two interacting CH3 domains are shown in light blue and blue colors. (**D**) Superposition of two C4n structures (shown in light salmon and brown colors) onto wildtype IgG4 Fc to illustrate the effects of the mutations at the CH3-CH3 interface.

The root-mean-square deviation (RMSD) of Cα atom coordinates between the final structure and the previously solved IgG4 Fc structure is only 0.53Å. This high degree of similarity renders these molecules as nearly identical. However, the CH3-CH3 interface of a conventional Fc homodimer was not observed. The conformation of the last 15 amino acids of in the monomeric Fc structure deviates substantially from the wildtype Fc. This phenomenon is due to the presence of Zn^2+^ ions in the crystallization solution (**S1 Fig**). The final model contains 1679 protein atoms, 110 carbohydrate atoms, two Zn ions, one glycerol molecule and 81 solvent molecules (**Fig 4A**). The peptide around the glycosylation site N297 spanning amino acids 290 through 300 and the glycans themselves appear somewhat less structured compared to the rest of the molecule (**Fig 4B**). The modeled biantennary carbohydrate moiety contains the core N-acetyl-D-glucosamine (GlcNAc or NAG) residue followed by another GlcNAc, followed by β-D-mannose (BMA) that splits the single carbohydrate chain into two chains. Each of these chains contains α-D-mannose (MAN) followed by GlcNAc, galactose (GAL), visible in one chain, and possibly a terminal sialic acid which is not visible in either of the chains. The core GlcNAc also has an α-L-Fucose residue attached.

The CH3-CH3 interface of a wildtype IgG4 Fc is illustrated in **Fig 4C**. To disrupt the interaction between the two CH3 domains, we had selected 5 interface residues, L351, S354, T366, F405 and Y407, for our mutagenesis library. Although P395 is not at the interface, it was included in the library design to provide for possible backbone relaxation necessary to arrive at a stable monomeric Fc [17]. Our X-ray crystal structure for C4n confirmed a monomeric Fc devoid of CH3-CH3 dimerization. To understand the impact of the mutations in C4 or C4n that resulted in the monomer formation, the structure of the monomeric Fc was superimposed with each of the two chains of the wildtype IgG4 Fc (**Fig 4D**). It was revealed that the mutations introduced in C4 or C4n are indeed incompatible with Fc homodimer formation. The mutation T366R would have caused steric clashes with T366R and L351F of the opposing polypeptide chain. Similarly, F405R would have caused serious clash with the K392 side chain from the opposite Fc chain. The Y407E mutation removes the aromatic stacking interaction from the two tyrosine side chains in that position, favorable for dimerization. In addition, the glutamic acid side chain in position Y407E would have caused clashes with the same amino acid in the opposing chain.

### Monomeric Fc binding to Fc receptors

We compared apparent binding affinities of C4 and C4n, along with Fc controls, to human FcRn. As expected, C4 and C4n showed no measurable binding to FcRn at pH 7.4 (data not shown). Using a steady-state binding analysis, we determined that at pH 6.0, C4 demonstrated similar FcRn binding (370 nM) compared to IgG4 wildtype Fc and IgG1 mAb controls (**Table 3**). This showed that specific mutant combinations could generate the desired structural fine-tuning to retain FcRn binding comparable to a wildtype IgG. We further investigated whether the FcRn binding affinity was augmented by the YTE mutations built into the library template. We found that the FcRn affinities of C4 and C4n differ by about 10-fold, similar to the difference in FcRn binding of IgG1 and IgG1 YTE.

**Table 3:** Equilibrium binding of Fc variants to human FcRn in avidity binding format.

Construct	Apparent K_D_ at pH 6
	*(nM)*
C4n	3560
C4	370
Onart-Fab-C4	160
Onart-scFv-C4	230
IgG4 Fc	140
IgG1	270
IgG1 YTE	39

We also assessed the binding of C4 to other Fc interacting proteins. We observed the expected lack of binding to the FcγRI and FcγRIII receptors (data not shown), consistent with structural evidence that the lower hinge regions of both Fc chains are needed for FcγR interaction [35, 36]. However, to confirm the proper folding of the lower hinge region, we used a mutant form of the enzyme IdeS, known to bind to the lower hinge, and found that IdeS retained low micro-molar range binding affinity (3 μM) to C4, comparable to wildtype Fc. This supports the structural data that the lower hinge regions of our monomeric Fc and wildtype Fc are similar in conformation.

### Monomeric Fc-fusion proteins

**Fig 5:**
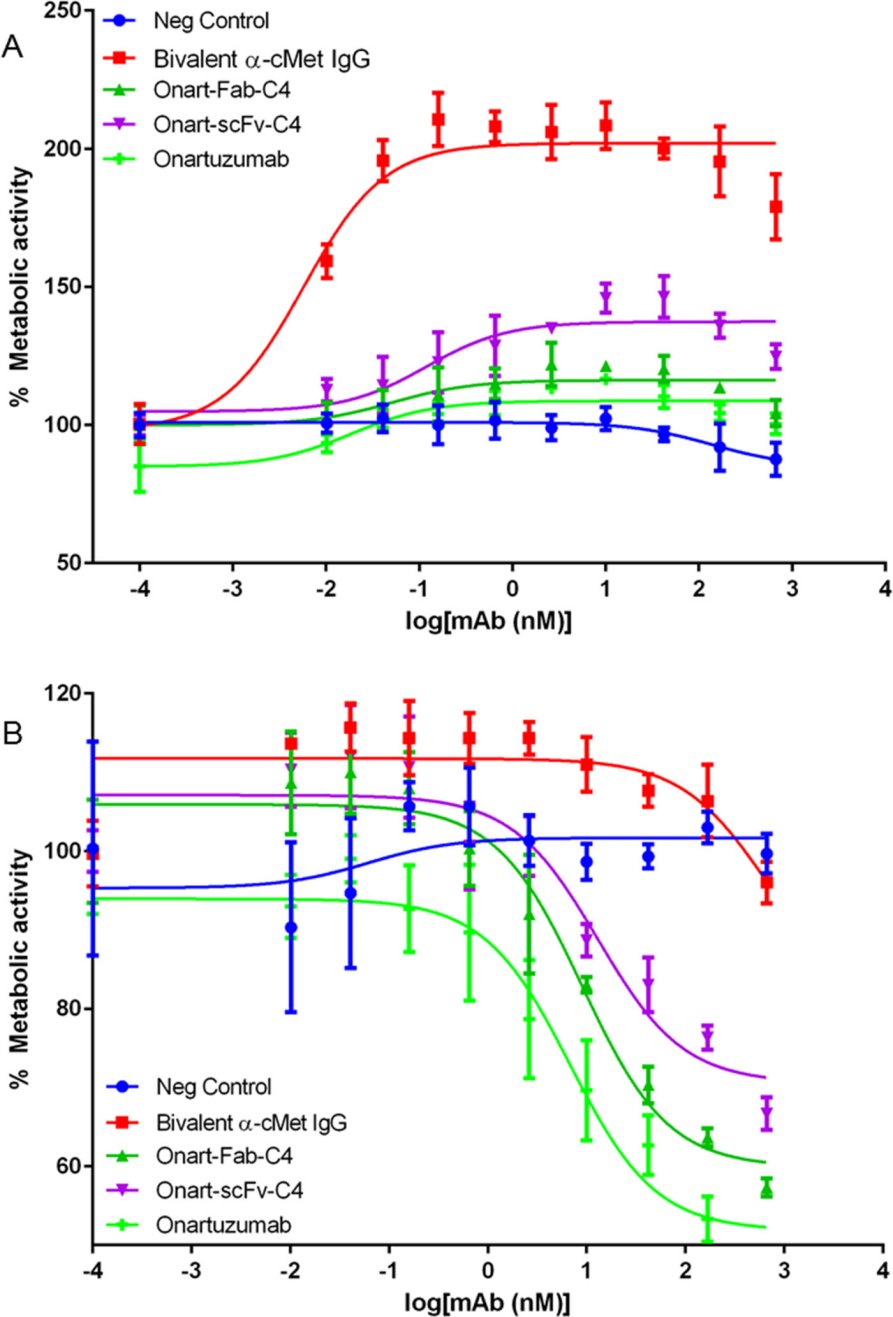
Growth inhibition activities of anti-cMet-C4 fusion proteins and controls. Lovo cells were treated with Onart-Fab-C4 (dark green), Onart-scFv-C4 (light purple), one-armed heterodimeric antibody Onartuzumab (light green) [6], bivalent anti-cMet IgG (red) and negative control antibody (blue) in triplicates and cell viability was measured by CellTiter-Glo assay after a 72-hour incubation. (**A**) Without ligand HGF treatment, there was a marked agonist effect when cells were exposed to bivalent anti-cMet IgG, an effect mostly absent in monovalent anti-cMet constructs. (**B**) With ligand treatment, anti-cMet targeting was shown to inhibit cell growth. Monomeric Fc fusions, Onart-Fab-C4 and Onart-scFc-C4 showed similar inhibitory effects as Onartuzumab.

To demonstrate that C4 can be used as a platform for monomeric Fc-fusion proteins, we generated C4 fusions with the Fab and scFv fragments of the anti-cMet antibody Onartuzumab [6]. cMet, a known cancer cell surface protein, is a member of the tyrosine kinase receptor family known to require monovalent targeting to avoid receptor agonism caused by receptor dimerization from bivalent antibodies. The C4-fusion proteins carrying either the Fab arm or the scFv arm of Onartuzumab retained binding to FcRn comparable to wildtype Fc (**Table 3**). Onart-Fab-C4 and Onart-scFv-C4 had high binding affinity to cMet, 0.5nM and 0.7nM, respectively. SEC-MALS demonstrated that these fusion constructs retained monomeric conformation (**Table 1**). In cell growth inhibition assays with cMet-expressing Lovo cells, there was a marked agonist effect when cells were exposed to bivalent anti-cMet IgG, which is mostly absent in monovalent anti-cMet constructs (**Fig 5A**). This agonist effect due to the bivalent nature of anti-cMet illustrates a well-documented phenomenon in membrane protein targeting in literature and has limited the therapeutic uses of bivalent IgGs in certain receptor targeting contexts [37]. On the other hand, when the cells were stimulated with HGF both Onart-Fab-C4 and Onart-scFv-C4 molecules showed potent cMet-specific cell growth inhibition activity, similar to the monovalent heterodimeric Onartuzumab (**Fig 5B**).

### *In vivo* PK studies

**Fig 6:**
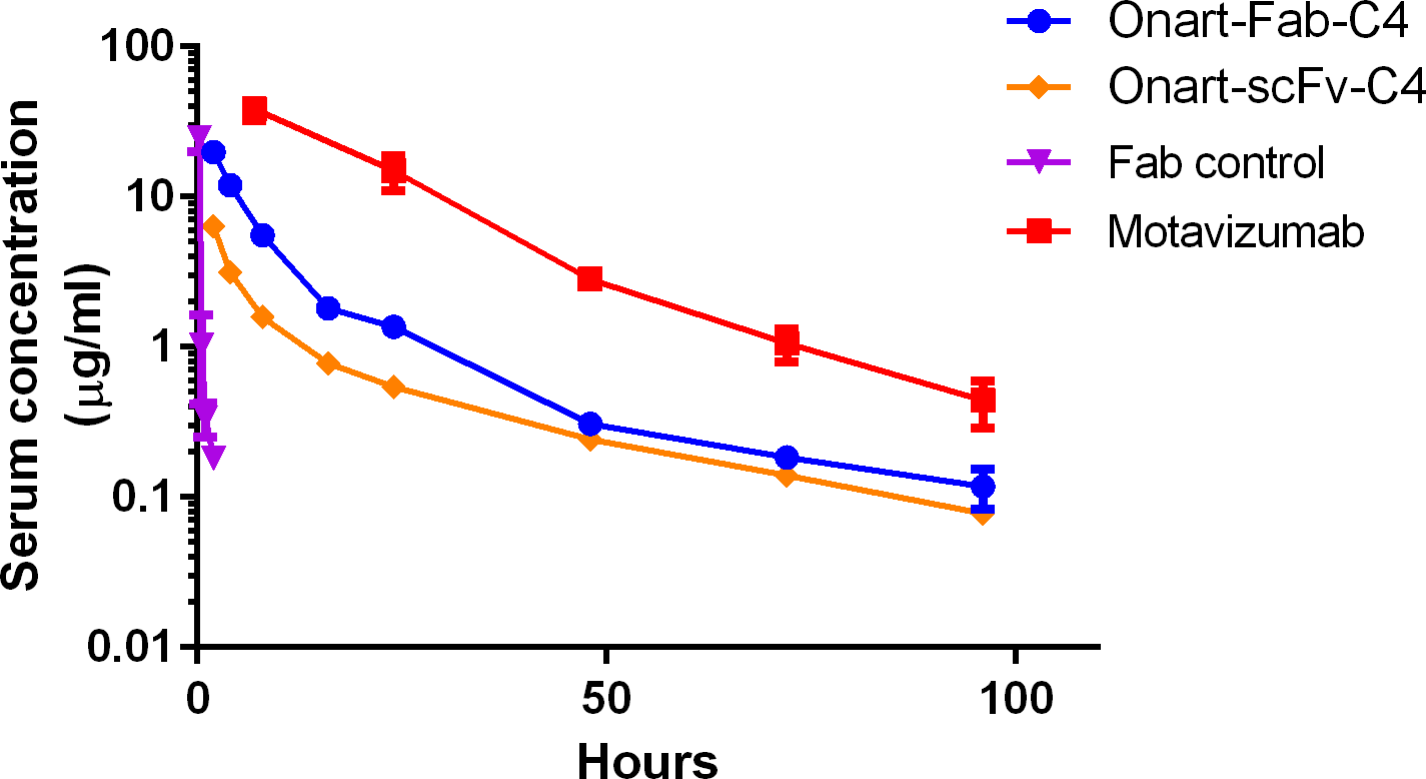
Pharmacokinetic profiles of monomeric fusion proteins in hFcRn transgenic mice. Serum clearance curves are plotted for a wild-type IgG control (Motavizumab, red), Onart-Fab-C4 (blue), Onart-scFv-C4 (orange) and Fab control (purple) in hFcRn transgenic mice. *Error bars*, SE (standard error). C4 significantly extended the serum half-life of the fusion proteins. Compared to a wildtype IgG control, C4 fusions had an initial faster distribution clearance, possibly attributed to enhanced tissue distribution (V_ss_, Table 4) and then achieved similar serum half-life (T_1/2_, Table 4).

To demonstrate that FcRn binding affinity of C4 can lead to improved serum half-life of a monovalent fusion protein, we carried out *in vivo* pharmacokinetic studies of the Onart-Fab-C4 and Onart-scFv-C4 fusion proteins in human FcRn transgenic mice. The mice were dosed with 2.5 mg/kg of fusion proteins, and serum protein concentrations were determined by ELISA. **Table 4**summarizes the relevant PK parameters. Compared to a Fab molecule alone, the monovalent Fc fusion proteins showed significant improvement in their PK properties (**Fig 6**), as manifested by 10-20-fold reduction in clearance (CL) and about 40-fold increase in terminal half-life (T_1/2_). This indicates that the C4-mediated human FcRn binding contributes to enhanced protein recycling. Compared to the wildtype IgG control, both monomeric Fc fusion proteins had a similar half-life of ~20 hours despite having 3-10-fold higher clearance. This was because the fusion proteins had a much larger volume of distribution (V_ss_), probably as a result of having smaller molecular weights compared to IgG.

**Table 4:** *In vivo* mouse pharmacokinetic analysis of monomeric Fc-fusion proteins.

Construct	Dose	C_max_	AUC_INF_	CL	T_1/2_	V_ss_
	(mg/kg)	(μg/mL)	(hr*μg/mL)	(mL/hr/kg)	(hr)	(mL/kg)
Fab	2.5	25 ± 5	8 ± 1	327 ± 58	0.6 ± 0.07	122 ± 28
Motavizumab	2.5	38 ± 6	877 ± 84	3 ± 0.3	18 ± 4	58 ± 9
Onart-Fab-C4	2.5	20 ± 2	160 ± 4	16 ± 0.4	21 ± 8	228 ± 49
Onart-scFv-C4	2.5	10 ± 7	92 ± 56	33 ± 15	24 ± 6	740 ± 410

PK parameters were determined by non-compartmental analysis using model 201. C_max_: peak concentration; AUC: the area under concentration; time curve: T_1/2_: terminal half-life; CL: clearance; V_ss_: volume in steady state.

## Discussion

We have generated a monomeric Fc via a combination of rational design and *in vitro* evolution approaches. We used MALS and analytical ultracentrifugation analyses, in addition to size-exclusion chromatography, to characterize the monomeric Fc and used the crystal structure to demonstrate that our IgG4 based monomeric Fc exhibits an IgG Fc-like structure. The unique combination of these mutations, L351F, T366R, P395K, F405R and Y407E, was crucial in forming an Fc monomer with good monodispersity, even though other mutations of the same CH3-CH3 interface positions have been reported previously [12, 13, 17]. More importantly, using the IgG4-YTE based monomeric Fc, we have demonstrated that a monomeric Fc-fusion protein can achieve equivalent serum half-life as an IgG. Taken together, our results suggest that the novel monomeric IgG4 Fc can be a promising therapeutic platform to extend the serum half-life of proteins in a monovalent format.

There had been reported challenges in developing monomeric Fc, such as monomer instability and reduced FcRn binding with disrupted CH3-CH3 dimer interface. For instance, there have been library-derived “monomeric” Fc mutants determined by SEC characterization that were later found to show only partial monomer formation when analyzed using light scattering [13, 14, 38]. Additionally, reduced FcRn binding has been reported by multiple studies attributing to the lack of binding avidity to FcRn [11, 12]. We incorporated the YTE mutations (M252Y/S254T/T256E) in the CH2 domain to enhance FcRn binding [15, 39] into our monomeric Fc template, and employed multiple biophysical and biochemical characterization methods to screen for stable Fc monomers with IgG-like binding affinity to FcRn. This approach also sidestepped possible stability issues with adding YTE retrospectively to an engineered Fc monomer. The designed thermal selection steps during phage selection were found crucial in the enrichment for stable FcRn-binding Fc monomers as without them, sequence enrichment was not achievable. Our IgG4-YTE based monomeric Fc, C4, demonstrated human FcRn binding affinity comparable to bivalent wildtype IgG4 or IgG1, which has not been previously demonstrated in a single monomeric Fc. Earlier studies had postulated that loss of avidity in Fc was the cause for reduced binding to FcRn [11, 12, 40]. Our data agreed with these observations, as we showed that C4 demonstrated weaker binding to FcRn by about 10-fold compared to IgG1 YTE (**Table 3**). However, C4 affinity to FcRn is comparable to wildtype IgG, showing that it is possible to overcome the loss of avidity by the gain in affinity using YTE for monomeric Fc. Our approach avoids the need for a tandem format to add avidity.

In this work, monomeric Fc-fusion proteins with anti-cMet Fab and scFv were generated and showed excellent *in vitro* activity and biophysical properties. Furthermore, these C4-based fusion proteins demonstrated IgG-like serum half-life, suggesting the FcRn mediated recycling was fully effective. For targets such as cMet, where antibody-mediated receptor dimerization has undesirable effects, the technology has a broad application in providing a therapeutic platform and research tools. It offers a simplified single-chain strategy to achieve monomeric and monovalent targeting, requiring only Protein A purification instead of additional purification processes necessary for monovalent heterodimeric Fc fusion proteins. PK analysis using human FcRn-transgenic mice demonstrated marked improvement of serum half-life over a Fab domain alone, which was cleared very rapidly because it is below the threshold of the renal glomeruli filtration size (~50–60 kDa) [41, 42]. In contrast, C4-fusion proteins, with only modest increase in size, resulted in over 10-fold slower clearance rate. More importantly, the serum half-life (T_1/2_) for Fab-C4 and scFv-C4 is similar to that of the IgG control. Furthermore, compared to IgG, the larger steady-state volume of distribution (V_ss_) suggests the monomeric Fc fusions may enhance tissue distribution. The role of reduced sizes of a half-life extending monomeric Fc fusion in tissue distribution can be further explored. Taken together, our novel monomeric IgG4 Fc construct and its fusion proteins demonstrated desirable *in vitro* activity and *in vivo* pharmacokinetics and could become a potential platform for generating monomeric Fc fusion proteins.

## Supporting Information

S1 Fig The C4n structure model revealed a crystallization artefact related to Zn^2+^-mediated chain swapping. The conformation of the last 15 amino acids in the monomeric Fc structure was changed substantially due to the presence of Zn^2+^ ions in the crystallization solution. As illustrated in (**A**), the 15 amino acids constituting the last β strand of Fc (in light salmon) were swapped with those from a symmetry related molecule (in green). (**B**) Detailed analysis showed that H433 and H435 have adopted a different position to coordinate a Zn^2+^ ion (shown in stereo). IgG4 Fc, in cyan, is superimposed with C4n structure in light salmon color, to show the extent of the conformational changes. (**C**) The tetrahedral coordination sphere of the Zn^2+^ ion is completed by H310 from the symmetry related molecule (in green) and a water molecule (W). (**D**) The electron density omit map of the chain swapping region is shown.
(DOCX)

## Abbreviations

Fc: fragment crystallizable
Fab: fragment antigen binding
scFv: single chain fragment variable
IgG: Immunoglobulin G
mAbs: monoclonal antibodies
CH3: heavy chain constant domain 3
SEC-MALS: size-exclusion chromatography coupled with multi-angle light scattering
PK: pharmacokinetics
hFcRn: human neonatal Fc receptor
FcγR: receptor for IgG Fc
YTE: M252Y/S254T/T256E
