# Supplementary material for: Generation and Characterization of an IgG4 Monomeric Fc Platform
Source: PLoS One. 2016 Aug 1;11(8):e0160345. doi: 10.1371/journal.pone.0160345 (PMC4968834; doi:10.1371/journal.pone.0160345)

**Supporting Information**

**S1 Figure**. The C4n structure model revealed a crystallization artefact related to Zn^2+^-mediated chain swapping. The conformation of the last 15 amino acids in the monomeric Fc structure was changed substantially due to the presence of Zn^2+^ ions in the crystallization solution and the absence of a second Fc chain that stabilizes the positions of the last β strand in the conventional Fc. As illustrated in (**A**), the 15 amino acids constituting the last β strand of Fc (in light salmon) were swapped with those from a symmetry related molecule (in green). (**B**) Detailed analysis showed that H433 and H435 have adopted a different position to coordinate a Zn^2+^ ion (shown in stereo). IgG4 Fc, in cyan, is superimposed with C4n structure in light salmon color, to show the extent of the conformational changes. (**C**) The tetrahedral coordination sphere of the Zn^2+^ ion is completed by H310 from the symmetry related molecule (in green) and a water molecule (W). (**D**) The electron density omit map of the chain swapping region is shown.


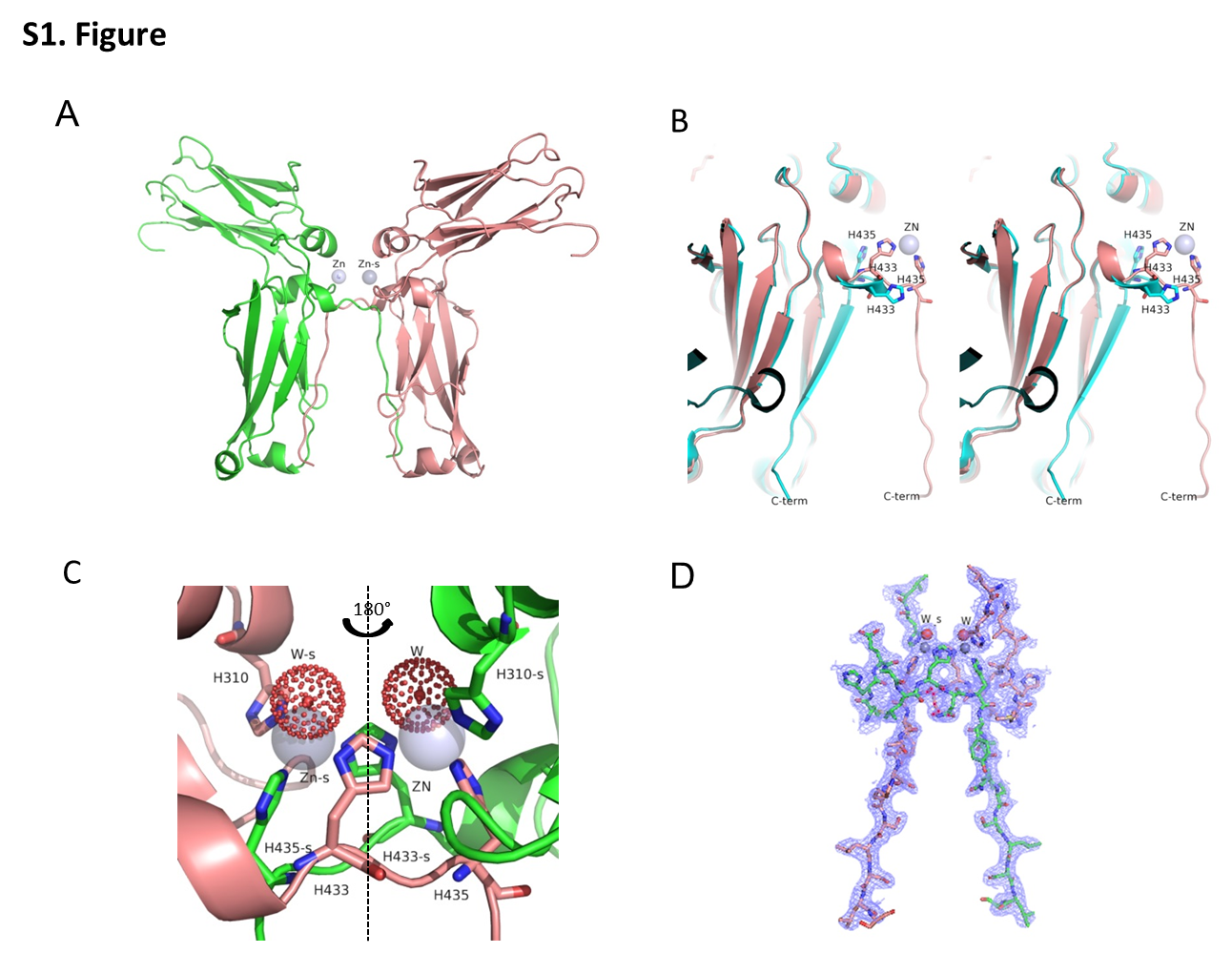

Supplement: S1 Fig — The conformation of the last 15 amino acids in the monomeric Fc structure was changed substantially due to the presence of Zn2+ ions in the crystallization solution. As illustrated in (A), the 15 amino acids constituting the last β strand of Fc (in light salmon) were swapped with those from a symmetry related molecule (in green). (B) Detailed analysis showed that H433 and H435 have adopted a different position to coordinate a Zn2+ ion (shown in stereo). IgG4 Fc, in cyan, is superimposed with C4n structure in light salmon color, to show the extent of the conformational changes. (C) The tetrahedral coordination sphere of the Zn2+ ion is completed by H310 from the symmetry related molecule (in green) and a water molecule (W). (D) The electron density omit map of the chain swapping region is shown. (DOCX) [file pone.0160345.s001.docx]
